# Coexistence of primary mediastinal MALT lymphoma and multiple myeloma like POEMS syndrome: A case report and literature review

**DOI:** 10.1097/MD.0000000000032801

**Published:** 2023-01-27

**Authors:** Shangjin Yin, Kuangguo Zhou, Zhiqiong Wang, Duanhao Gong, Wei Huang

**Affiliations:** a Department of Hematology, Tongji Hospital, Tongji Medical College, Huazhong University of Science and Technology, Wuhan, Hubei, China; b Department of Hematology, The Second Affiliated Hospital of Guizhou University of Traditional Chinese Medicine, Yunyan District, Guiyang City, Guizhou Province, China.

**Keywords:** light chain-restricted plasmacytic differentiation, mediastinal MALT lymphoma, multiple myeloma, treatment, case report

## Abstract

The coexistence of the extranidal marginal zone lymphoma (MZL) of mucosa–associated lymphoid tissue (MALT) and multiple myeloma (MM) is an exceedingly rare situation. The rare situation precludes any evidence-based guidelines for MZL or MM.

**Patient concerns and diagnoses:**

We presented a unique case of the coexistence of primary mediastinal MALT lymphoma and MM like polyneuropathy, organomegaly, endocrinopathy, M-protein, skin syndrome.

**Interventions and outcomes:**

The patient was first diagnosed with polyneuropathy, organomegaly, endocrinopathy, M-protein, skin syndrome in the department of neurology, then MM in the department of hematology, and the mediastinal MALT simultaneously coexisting with MM was found by biopsy in the department of thoracic surgery. The patient received combination therapy with rituximab and bortezomib followed by lenalidomide maintenance. To understand MZL lymphoma with plasmacytic differentiation better, we analyzed cases of MZL lymphomas with plasma cell neoplasms. Most of these cases were MZL lymphomas with light chain-restricted plasmacytic differentiation. The lymphomas relapsed with plasma cell neoplasms or transformed into plasma cell neoplasms after anti-lymphoma therapy.

**Lessons:**

The case demonstrated clinical complexity and the importance of the detailed assessment. The case and literature review demonstrated the value of detecting light chain-restricted plasmacytic differentiation for the treatment of MZL lymphoma with rituximab plus lenalidomide or bortezomib.

## 1. Introduction

The marginal zone lymphoma (MZL) accounts for approximately 5% to 15% of all non-Hodgkin lymphomas. MZLs have entities: the extranidal MZL of mucosa–associated lymphoid tissue (MALT), the splenic MZL, and the nodal MZL. MALT lymphoma is the most common form of MZL. Primary mediastinal lymphoproliferative malignancies are rare, accounting for less than 5% of all mediastinal malignancies.^[1]^ Primary mediastinal MALT lymphomas are very rare.

Multiple myeloma (MM) is a tumor that generally affects elderly individuals. MM accounts for 10% to 15% of all hematologic malignancies.^[2]^ The progression of MM is accompanied by numerous complications, which are typically summarized by the acronym (hypercalcemia, renal failure, anemia, and bone lesions). Peripheral neuritis at the time of diagnosis is uncommon in patients with MM. In patients with IgG or IgA M-protein, the incidence of peripheral neuropathy is lower than in patients with the IgM M-protein.^[2]^ POEMS (polyneuropathy, organomegaly, endocrinopathy, monoclonal gammopathy, and skin changes) syndrome is a rare plasma cell disorder, which is characterized by demyelinating peripheral neuropathy. The first-episode peripheral neuritis is very common in patients with POEMS syndrome.

We reported 1 case of coexistence of primary mediastinum MALT lymphoma and MM like POEMS syndrome, which presented with first-episode peripheral neuritis. To understand MZL lymphoma with plasmacytic differentiation better, we analyzed cases of MZL lymphomas with plasma cell neoplasms. The analysis suggested that rituximab combined with lenalidomide or/and bortezomib might be a more reasonable treatment option for MZL lymphoma with light chain-restricted plasmacytic differentiation.

## 2. Case report

**Figure 1.**
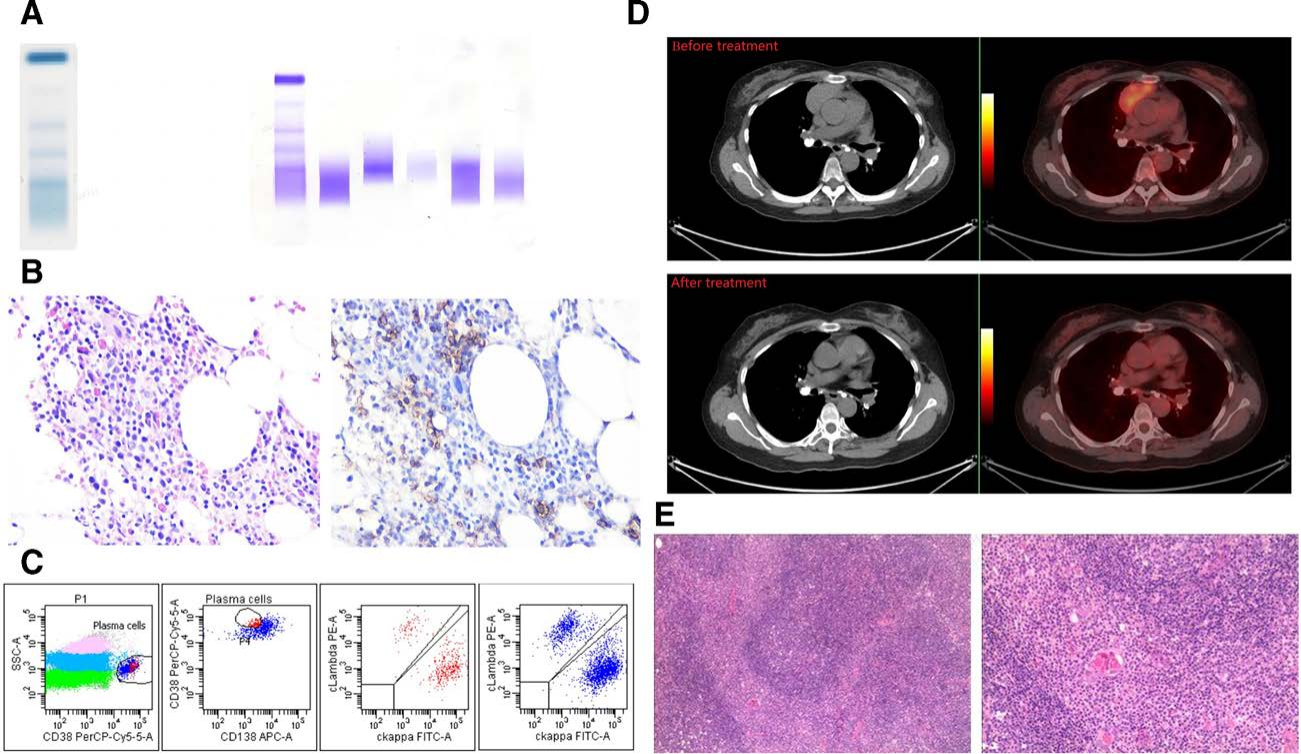
Data from the patient before and after treatment. (A) Monoclonal gamma globulin identification; (B) bone marrow biopsy and immunohistochemistry on bone marrow biopsy (CD38 positive); immunohistochemistry results: MPO (granulocyte)+;CD42b(megakaryocyte)+;CD3(very few) +;CD20 (very few) +;plasma cells CD38 +; CD138 +; bcl-2-;κ +;λ-;CD56-; (C) flow immunophenotype of bone marrow plasma cells with phenotypic abnormalities (CD38+; CD138+; CD229+; c kappa+; BCMA+; CSI+; CD45-; CD19-; CD56-; CD20-; clambda-; CD13-; CD33-; CD117-; HLA-DR-); (D): PET-CT before and after treatment; (E): biopsy of the mediastinal masses, MALT lymphoma with plasmacytic differentiation The immunophenotype of lymphoma cells: CD20 (+, positive control+), CD19 (+), CD22 (+), CD79a (+), PAX-5 (+), PAX8 (+), BCL2 (+), MNDA (+), CD21 and CD35 (a little +, FDC networks+), CD3 (-), CD5 (-), CD43 (-), CD10 (-), BCL6 (-), IgD (-), CD1a (-), TDT (-), CD117 (-,positive control +),P53 (partial +,wild-type), Ki-67 LI:about 5%; the immunophenotype of plasma cells: MUM1 (+), κ (+), λ (a little +,monoclonal) ; EBER CISH (-, positive control +);IgM (-). MALT = the extranidal marginal zone lymphoma of mucosa - associated lymphoid tissue.

A 51-years-old female patient was admitted to the department of neurology of our hospital because of progressive numbness in extremities with tingling and weakness for 4 years in April 2021. Her past medical history included a traumatic lumbar fracture in 2019. The neurological physical examination showed slightly decreased muscle strength of the lower extremities, hypoalgesia of the lower extremities, positive pussep sign in the right lower limb, and negative pathological reflexes such as the Babinski reflex. The physical examination showed no hepatosplenomegaly, no superficial lymph node swelling, and no bone pain. The electromyography showed bilateral upper and lower limb neurogenic damage (involving motor fibers, nerve roots, and myelin sheath). The ultrasound revealed pericardial effusion. The laboratory data showed anemia [89 g/L (normal: 115–150 g/L)], a high serum creatinine level [236 umol/L, (normal: 45–84 umol/L)] with proteinuria, high serum globulin [62.5 g/L, (normal: 20–35 g/L)], abnormal erythrocyte sedimentation rate [108 mm/H (normal: 0–20 mm/H)]. The overall autoimmune workup revealed several positive autoantibodies such as antinuclear antibodies, anti-SS-A antibodies, anti-SS-B antibodies, and anti-Ro52 antibodies. The level of prolactin [51.76 ng/mL (normal: 2.74–19.64 ng/mL)] increased significantly. Cerebrospinal fluid tests were normal. Monoclonal gamma globulin identification showed IgA-κ M protein. Among them, the quantitative results from serum protein electrophoresis showed the absolute value of serum M protein was 9.8g/L (normal: 0 g/L). The quantitative results from serum immunofixation electrophoresis showed abnormal levels of IgA [19.10 g/L, (normal: 0.82–4.53 g/L)], IgG [30.9 g/L, (normal: 7.51–15.6 g/L)], IgM [2.3 g/L, (normal: 0.46–3.04g/L)], serum free light chain κ [161.3 mg/L, (normal: 3.30–19.40 mg/L)], serum free light chain λ[155.3 mg/L, (normal: 5.71–26.30 mg/L)], κ/λ[1.039, (normal: 0.26–1.65)] (Fig 1A). The level of beta-2 microglobulin [6.76 mg/L (normal: 0.8–2.2 mg/L)] increased significantly. The magnetic resonance imaging of the central nervous system was normal. According to the diagnostic criteria proposed by Dispenzieri^[3]^ in 2003, patients who met 2 major criteria and at least 1 minor criterion could be diagnosed with POEMS syndrome. The patient was transferred to the department of hematology because of a diagnosis of POEMS syndrome. The serum vascular endothelial growth factor (VEGF) was detected, and the level of VEGF was normal. Then bone marrow tests were carried out. Bone marrow biopsy revealed 10% monoclonal plasma cells (Fig 1B). The flow cytometric analysis of bone marrow revealed plasma cells with phenotypic abnormalities (CD38+, CD138+, CD229+, ckappa+, BCMA+, clambda-, CD45-, CD19-, CD56-, CD20-, CD13-, FMC33-, CD117-, HLA-DR-) (Fig 1C). The patient was diagnosed with multiple myeloma (R-ISS stage III) like POEMS syndrome. To accurately assess the disease, the patient had a PET-CT test. PET-CT showed a hypermetabolic mass in the anterior mediastinum (SUVmax6.1) (Fig 1D). We referred the patient to thoracic surgery to further clarify the mediastinal mass. She underwent a mediastinal mass biopsy under video-assisted thoracoscopy. The pathological diagnosis was MALT lymphoma with light chain-restricted plasmacytic differentiation (Fig 1E), the immunophenotype of lymphoma cells: CD20 (+, positive control +), CD19 (+), CD22 (+), CD79a (+), PAX-5 (+), PAX8 (+), BCL2 (+), MNDA (+), CD21 and CD35 (a little +, FDC networks +), CD3 (-), CD5 (-), CD43 (-), CD10 (-), BCL6 (-), IgD (-), CD1a (-), TDT (-), CD117 (-, positive control+), P53 (partial+, wild-type), Ki-67 LI (about 5%); the immunophenotype of plasma cells: MUM1 (+), κ (+), λ (a little +, monoclonal); EBER CISH(-, positive control +); IgM (-). No mutation of exon 5 of MYD88 was detected. The patient was eventually diagnosed with MALT lymphoma and MM like POEMS syndrome. In May 2021, the patient received 1 course of combination therapy (rituximab 375 mg/m^2^, day 1; bortezomib 1.3 mg/m^2^, day 1, 4, 8, 11; dexamethasone 20 mg, day 1, 2, 4, 5, 8, 9, 11, 12). The peripheral neuritis was significantly improved. Bone marrow biopsy and flow cytometric analysis showed no monoclonal plasma cells. The results from serum protein electrophoresis showed the absolute value of serum M protein was 1.5 g/L. The patient completed additional 4 courses of combination therapy (rituximab; bortezomib; dexamethasone). PET-CT showed there was no hypermetabolic tumor in the anterior mediastinum. Bone marrow biopsy and flow cytometric analysis showed negative minimal residual disease of monoclonal plasma cells. The patient continued to receive lenalidomide maintenance (25 mg day 1–21,28 days/a cycle).

## 3. Discussion

MM and POEMS syndrome belong to a rare class of blood neoplasms that affect the plasma cells, but their clinical manifestations are significantly different. POEMS syndrome often begins with peripheral neuritis, and these patients were often referred first to the neurology department because of peripheral neuritis. In addition to major criteria including peripheral neuritis and monoclonal plasma proliferative disorder, there were minor criteria of POEMS syndrome including sclerotic bone lesions, Castleman disease, organomegaly (splenomegaly, hepatomegaly, or lymphadenopathy), edema (edema, pleural effusion, pericardial effusion or ascites), endocrinopathy (adrenal, thyroid, pituitary, gonadal, parathyroid, pancreatic), skin changes (hyperpigmentation, hypertrichosis, plethora, hemangiomata, white nails), and papilledema.^[3]^ According to the diagnostic criteria proposed by Dispenzieri^[3]^ in 2003, patients who met 2 major criteria and at least 1 minor criterion could be diagnosed with POEMS syndrome. Our patient met 2 major criteria (peripheral neuritis and monoclonal plasma proliferative disorder) and 2 minor criteria (pericardial effusion and endocrinopathy), so the diagnosed of POEMS syndrome was reasonable. Peripheral neuritis at the time of diagnosis is uncommon in patients with MM. In patients with IgG or IgA M-protein, the incidence of peripheral neuropathy is lower than in patients with the IgM M-protein.^[2]^ MM often begins with hypercalcemia, renal failure, anemia, and bone lesions symptoms, so the patients were often referred first to the department of orthopedics or department of nephrology, besides the department of hematology. The difference is that the proportion of bone marrow plasma cells is significantly different between MM and POEMS syndrome, besides different symptoms. Bone marrow plasma cells ≥10% by CD138 immunohistochemistry screening for MM.^[2]^ Our case also met the diagnosis of MM. VEGF has been included as a diagnostic criterion for POEMS syndrome in recent years. The value of the VEGF test was to distinguish POEMS syndrome from chronic inflammatory demyelinating polyradiculoneuropathy.^[4]^ POEMS syndrome with the normal level of VEGF was reported.^[5]^ The level of VEGF in our case was normal, it was reasonable that the case was diagnosed as MM(IgA-κ) like POEMS syndrome.

Monoclonal plasma proliferative disorders are a common cause of paraproteinemia. In addition to plasma cell tumors, autoimmune diseases and B-cell lymphomas, especially marginal zone lymphomas and lymphoplasmacytic Lymphoma, are also responsible for paraproteinemia.^[6]^ Monoclonal plasma cells (usually found by bone marrow aspiration) are important pieces of evidence to rule out autoimmune diseases and B-cell lymphomas. B-cell lymphomas in bone marrow were characterized by monoclonal B cells detected by immunohistochemistry and flow cytometry. PET-CT test showed a hypermetabolic mass in the anterior mediastinum, which indicated the presence of a neoplastic lesion. The neoplastic lesion might reasonably be interpreted as plasmacytoma associated with MM before the pathological biopsy.

The increased monoclonal immunoglobulins and decreased synthesis of uninvolved immunoglobulins are important features of MM,^[2]^ but our patient had elevated levels of both monoclonal IgA and non-monoclonal IgG, which was not consistent with MM. Studies showed that MALT lymphomas frequently presented elevated levels of polyclonal immunoglobulins. Most patients of primary mediastinal MALT lymphoma had an autoimmune disease or hyperglobulinemia, and both increased serum autoantibody levels. The polyclonal serum immunoglobulin levels remained essentially unchanged after total surgical resection in all patients.^[7]^ So primary mediastinal MALT lymphoma could not be ruled out in our patient and the mediastinal mass biopsy was necessary. The pathological diagnosis from the mediastinal mass was MALT lymphoma with plasmacytic differentiation. The patient was eventually diagnosed with MALT lymphoma and MM like POEMS syndrome.

Clinically, MZL lymphoma first presenting as peripheral neuropathy was rarely reported. Through a literature search, we retrieved 4 cases of MZL lymphoma with first peripheral neuropathy, the serum M protein of these 4 cases was monoclonal IgM type.^[8–10]^ We presented the first report of the coexistence of primary mediastinal MALT lymphoma and MM like POEMS syndrome, which first presented with peripheral neuritis.

Not only are the clinical presentation and treatment different, but the tumorigenesis of MZL with plasmacytic differentiation and MM are also different. Chronic inflammation plays an important role in plasma cell differentiation and tumorigenesis. In up to 30% of patients diagnosed with MALT lymphoma, there was the feature of plasmacytic differentiation. Patients with plasmacytic differentiation had a significantly higher rate of extragastric MALT lymphoma.^[11]^ The plasmacytic differentiation in MALT lymphoma was from monoclonal B cells further stimulated by antigen. The monoclonal plasma cells in MM were derived from plasma cell transformation under various tumorigenic factors.

Through literature review, we summarized 7 patients with MZL lymphoma and plasma cell neoplasm (Table 1).^[12–18]^ Of these 7 patients, 1 patient was diagnosed with the coexistence of cervical nodal MZL lymphoma and MM (Lambda type) at the initial diagnosis,^[12]^ and the other 6 patients were diagnosed with plasma cell neoplasms after the anti-lymphoma chemotherapy. Of these 6 patients, 4 patients were diagnosed with plasma cell neoplasms at the time of lymphoma recurrence,^[13–16]^ and 2 patients were diagnosed with plasma cell neoplasms transformed from lymphomas.^[17,18]^

**Table 1:** Clinical data of 7 cases of marginal zone lymphoma with with plasma cell neoplasms collected from literature search.

case	Sex	Age (yr)	Type of MZL	Plasmacytic differentiation	Light chain restriction of plasma cell in lymphoma	Site of lymphoma involvement	Type of plasma cell neoplasm	Serum M protein	Cloning identification	Diagnosis sequence	Treatment	References
1	M	66	nodal MZL	yes	kappa	cervical and inguinal regions	MM	lambda type	NA	simultaneously	PAD	^[12]^
2	M	83	nodal MZL	yes	lambda	left cervical (submandibular) lymph node, bone marrow	MM	IgA (k)	the same clone	nodal MZL first, and then MZL recurred with MM	Rad first, then CHOP	^[13]^
3	M	80	MALT	yes	kappa	left eye	MM	IgG- kappa	two different clones	MGUS 5.5 yr ago, MALT 4 yr ago, then MALT recurred with MM	2-CdA + R, then Rad + MP	^[14]^
4	F	73	MALT	yes	lambda	small intestinal	plasma cell leukemia	kappa	the same clone	MALT first, and then MALT recurred with plasma cell leukemia	R-CHOP, BR, then VCD	^[15]^
5	M	59	MALT	NA	NA	pulmonary	plasmacytoma (pleural effusion)	IgM-kappa	the same clone	MALT first, then MALT progression with plasmacytoma	R-COP, then no Chem	^[16]^
6	M	82	MALT	yes	kappa	stomach and lung	plasmacytoma (right lung)	IgM- kappa	the same clone	MALT first, then MALT transformed to plasmacytoma	R first, then oral CTX	^[17]^
7	F	39	MALT	yes	NA	pulmonary	plasmacytoma (nasal cavity)	IgG-kappa	the same clone	MALT first, then cavity plasmacytoma	R first, then Rad	^[18]^

BR = bendamustine + Rituximab, Chem = chemotherapy, CHOP = cyclophosphamide + doxorubicin hydrochloride + vincristine Sulfate+ prednisone, CTX = cyclophosphamid, 2-CdA = 2’-Chlorodeoxyadenosine, MM = multiple myeloma, MP = melphalan+ prednisone, MZL = marginal zone lymphoma, MALT = the extranodal MZL of mucosa - associated lymphoid tissue, MGUS = Monoclonal gammopathy of undetermined significance, NA = not clear in the article, PAD = bortezomib+ liposomal adriamycin+ dexamethasone, R = rituximab, Rad = Radiation, VCD = bortezomib+ cyclophosphamide+ dexamethasone.

Among the 7 cases reviewed, all the other 6 cases had plasmacytic differentiation, except for case 5 with incomplete data. Of these 6 cases, all the other 5 cases had light chain restriction of plasma cells in lymphoma pathology, except for case 7 with incomplete data. This suggested that MZL lymphomas with light chain-restricted plasmacytic differentiation might transform into plasma cell neoplasms after rituximab-based anti-lymphoma chemotherapy.

In 6 of the 7 cases, clones were identified. In 5 of the 6 cases, monoclonal plasma cells and B cells were derived from the same clone.^[13,15–18]^ In case 3, monoclonal plasma cells and B cells were derived from different clones. Case 3 had had monoclonal plasma cells for 4 years at the time of diagnosis of MALT lymphoma, MGUS progressed to MM at the time of lymphoma recurrence, and clonal identification showed that the 2 B-cell malignancies were clonally unrelated.^[14]^ The development of MM is a long-term chronic process. Monoclonal gammopathy of undetermined significance (MGUS) is the asymptomatic precursor of MM. The cumulative probability of MGUS progression to MM or related disorders was 10% and 25% at 10 years and 20 years, respectively.^[19]^ Another report showed that MGUS was present for 13 years at the time of diagnosis of MALT lymphoma,^[20]^ despite MGUS did not transform into MM.

Bortezomib and lenalidomide are currently the first-line therapies for MM. Bortezomib and lenalidomide were also generally used to treat B-cell lymphoma. In 1 phase II study of single bortezomib in previously untreated MLAT lymphoma, the overall response rate was 80%; the complete remission rate was 43% and the partial response rate was 37%.^[21]^ The treatment of bortezomib combined with rituximab and dexamethasone for lymphoplasmacytic lymphoma resulted in an overall response rate of 85% and a 3-year survival rate of 81%.^[22]^ In 1 phase II study of lenalidomide plus rituximab in previously untreated MLAT lymphoma, the overall response rate was 93% with 70% attaining CR, the median progression-free survival was 59.8 months and 5-year overall survival was 96% at a median follow-up of 75.1 months.^[23]^ Lenalidomide alone was used to treat MALT lymphoma and had an overall response rate of 61%.^[24]^ The results of the pharmacoeconomic analysis showed that it was better clinical effectiveness and better cost-effectiveness for lenalidomide plus rituximab treating adults with follicular lymphoma or MZL lymphoma, compared with the conventional chemotherapy regimen such as R-CHOP/R-CVP.^[25]^ Although these studies yielded some positive results, these studies did not pay attention to MALT lymphomas with light chain-restricted plasmacytic differentiation.

Of the 7 patients in our analysis, case 1 of the coexistence of lymphoma and MM simultaneously was treated with a bortezomib-based chemotherapy regimen (bortezomib, liposomal adriamycin + dexamethasone).^[12]^ The lymphoma and MM were effectively controlled simultaneously. Our patient was treated with combination therapy of bortezomib + rituximab + dexamethasone followed by sequential lenalidomide maintenance, and the disease remains stable without progression. This suggested that rituximab combined with lenalidomide or/and bortezomib might be a more reasonable treatment option for MZL lymphoma with light chain-restricted plasmacytic differentiation. The rituximab combined with lenalidomide or/and bortezomib might not only be beneficial to improve the efficacy of MZL lymphoma with light chain-restricted plasmacytic differentiation but also to prevent the progression of malignant plasma cell clones into MM.

In brief, we report a rare case of coexistence of MALT lymphoma and MM like POEMS syndrome firstly presented with peripheral neuritis, which demonstrated clinical complexity and the importance of biopsy. Given our case and literature analysis, we suggested that rituximab combined with lenalidomide or/and bortezomib might be a more reasonable treatment option for MZL lymphoma with light chain-restricted plasmacytic differentiation. This showed the value of detecting light chain-restricted plasmacytic differentiation for the treatment of MZL lymphoma with rituximab plus lenalidomide or bortezomib.

## Acknowledgments

We thank the patient and their families.

## Author contributions

**Conceptualization:** Wei Huang.

**Data curation:** Shangjin Yin.

**Formal analysis:** Kuangguo Zhou.

**Investigation:** Duanhao Gong.

**Methodology:** Zhiqiong Wang, Duanhao Gong.

**Project administration:** Wei Huang.

**Resources:** Kuangguo Zhou.

**Software:** Duanhao Gong.

**Validation:** Zhiqiong Wang.

**Visualization:** Zhiqiong Wang.

**Writing – original draft:** Shangjin Yin.

**Writing – review & editing:** Wei Huang.
